# Single-Step Sampling Approach for Unsupervised Anomaly Detection of Brain MRI Using Denoising Diffusion Models

**DOI:** 10.1155/ijbi/2352602

**Published:** 2024-12-19

**Authors:** Mohammed Z. Damudi, Anita S. Kini

**Affiliations:** Department of Computer Science & Engineering, Manipal Institute of Technology, Manipal Academy of Higher Education (MAHE) 576104, Manipal, Karnataka, India

## Abstract

Generative models, especially diffusion models, have gained traction in image generation for their high-quality image synthesis, surpassing generative adversarial networks (GANs). They have shown to excel in anomaly detection by modeling healthy reference data for scoring anomalies. However, one major disadvantage of these models is its sampling speed, which so far has made it unsuitable for use in time-sensitive scenarios. The time taken to generate a single image using the iterative sampling procedure introduced in denoising diffusion probabilistic model (DDPM) is quite significant. To address this, we propose a novel single-step sampling procedure that hugely improves the sampling speed while generating images of comparable quality. While DDPMs usually denoise images containing pure noise to generate an original image, we utilize a partial diffusion approach to preserve the image structure. In anomaly detection, we want the reconstructed image to have a structure similar to the original anomalous image, so that we can compare the pixel-level difference between them in order to segment the anomaly. The original DDPM algorithm suggests an iterative sampling procedure where the model slowly reduces the noise, until we have a noise-free image. Our single-step sampling approach attempts to remove all the noise in the image within a single step, while still being able to repair the anomaly and achieve comparable results. The output is a binary image showing the predicted anomalous regions, which is then compared to the ground truth to evaluate its segmentation performance. We find that, while it does achieve slightly better anomaly masks, the main improvement is in sampling speed, where our approach was found to perform significantly faster as compared to the iterative procedure. Our work is mainly focused on anomaly detection in brain MR volumes, and therefore, this approach could be used by radiologists in a clinical setting to find anomalies in large quantities of brain MRI.

## 1. Introduction

Generative artificial intelligence (AI) describes algorithms that can be used to create new content, including audio, code, images, text, simulations, and videos. Generative modeling is an unsupervised form of machine learning where the model learns to discover the patterns in input data. Using this knowledge, the model can generate new data on its own, which is related to the original training dataset. In this way, they may be used to generate new data points that look similar to one in the training data, using past data points. For example, models that predict the next word in a sequence are typically generative models. Some examples of generative models that are currently undergoing research and are being used in the real world are generative adversarial networks (GANs), variational autoencoders (VAEs), and diffusion models. Each of these generative models can produce high-quality images but they also have their own limitations.

Diffusion models specifically are more common in the image generation space and are being used in most of the AI developments that have taken place over the past few months in the world of image generation, such as the open-source implementation of stable diffusion which is based on latent diffusion models (LDMs) [1], and other implementations such as DALL-E, Imagen [2], and Midjourney. All these techniques use slight variations of diffusion models to reach the same end goal, to generate new images. Diffusion models are used in a variety of image processing tasks such as conditional image generation, text-to-image synthesis, image-to-image translation, image superresolution, and image inpainting.

For most of the past decade, GANs [3] and VAEs [4] have been the most popular generative models being used in the real world and had found various practical applications across different domains. But when denoising diffusion models [5] were first introduced in 2020 and shown to create high-quality images, researchers quickly hopped on this new technique and new developments were being made at an extremely fast pace, and they were eventually found to perform even better than GANs [6]. It introduced us to a whole new world of possibilities. Companies such as stability AI made its implementation (stable diffusion) open source and made its trained models public for anyone to experiment with, due to which anybody in the world could have access to such powerful tools. This made deep learning research in diffusion models even more prominent.

The original paper by Sohl-Dickstein et al. [7] introduced the idea of using diffusion to the field of machine learning, which came originally from statistical physics. The essential idea is to apply a lot of noise to an image and destroy the image signal, after which a neural network can be used to remove this noise. When the neural network is learnt properly, we can start off with a completely random noised image and let the model remove noise iteratively until we have a completely original image. The authors showed that during the forward diffusion process, the image will converge to pure noise which follows the noise distribution. The reverse diffusion process includes learning the reverse process to go from pure noise to an actual image, which involves a neural network learning to remove noise from an image step by step during the training process. During sampling, we can simply give the model an image consisting of random noise sampled from a normal distribution and let the model gradually remove the noise until we have a clear image. The reason this operation is performed in a stepwise manner is that this method would not be tractable and lead to much worse outcomes. Learning in this framework involves estimating small perturbations to a diffusion process, which is more tractable than explicitly describing the full distribution with a single function.

Diffusion models are more stable than GANs, which are subject to mode collapse, where they only represent a few modes of the true distribution of data after training, while diffusion models are more faithful to the data and tend to be much slower, iterative, and a much more guided process. They are capable of generating samples from complex data distributions with superior mode coverage [8] over GANs and VAEs. They also have fewer hyperparameters and are relatively straightforward to tune during the training process, whereas hyperparameter tuning for GANs can be a challenging and time-consuming process. They also do not require very large datasets to learn the structure of images, avoiding the limitations common in GAN-based approaches.

The one disadvantage with diffusion models is that due to its iterative, guided process, it has an extremely slow sampling speed as compared to its counterparts. When it comes to inference time, even though it would create better and more diverse and high-quality images, its sampling speed is one factor that holds it back from being used in real-world time-sensitive scenarios. Depending on the type of data being generated and the compute resources available, it could take a significant amount of time.

The initial frameworks for anomaly detection were based on the foundational concept that a model learns to accurately reconstruct nominal data, while failing to reconstruct anomalies. These are called reconstruction-based methods for anomaly detection. Typically, models learn to generate nonanomalous (healthy) images or regions of interest from anomalous data, after which abnormalities are identified by assessing whether they lie outside the manifold of the learned representation or by comparing the generated and original images in pixel space. Various approaches for anomaly detection using generative models have been explored. Lu and Xu in [9] applied VAE to detect anomalies in skin disease images. Pidhorskyi et al. in [10] used an adversarial autoencoder to effectively compute the likelihood of a sample generated by the inlier distribution. GANomaly [11] used a conditional GAN to perform semisupervised anomaly detection. AnoGAN [12], which was among the pioneering GAN-based methods for anomaly detection, uses a scoring mechanism that considers discrepancies in both pixel-space and discriminator features between a test image and an iteratively refined generator approximation. However, this refinement process involves numerous backpropagation iterations, leading to slow inference times. In the medical domain, denoising diffusion models have been used to detect brain tumors [13]. All methods that previously used diffusion models for anomaly detection previously used the default Gaussian noise as the noise function.

In this paper, we propose a single-step sampling (SSS) procedure, which works particularly well in cases where we use diffusion models for one-class anomaly detection. This technique was tested specifically on brain MRI and was found to be successful at detecting large anomalous regions, where the model is trained on healthy, nonanomalous brain imaging data. The idea is for the diffusion model to take in an anomalous brain image and repair it by reconstructing it as a healthy brain image, which can then be used to find the difference between the original and reconstructed images. This difference will then be detected as the anomaly. This method of anomaly detection could be used by radiologists in a clinical setting to find anomalies in large quantities of brain MRI. The main contributions of this work are as follows:

1. To develop a SSS approach with partial diffusion and simplex noise to construct original images using a single reverse diffusion step.
2. Introduce an alternative inference technique where we sample across multiple timesteps and average their corresponding reconstructions.

## 2. Related Works

Diffusion models have become popular for image generation and achieve competitive results to the usual state-of-the-art GANs. Especially in the generative art field, diffusion models have achieved amazing results for text-to-image synthesis. Recently, these models have been shown to generate incredibly detailed results even in video generation tasks.

Denoising diffusion probabilistic models (DDPMs) by Ho, Jail, and Abbeel [5] introduced a few ground-breaking changes, which lead to a huge improvement in image quality. This is one of the most relevant papers in recent times, as it revolutionized the development of diffusion models. The authors suggested that rather than predicting the image signal, we could simply have the neural network predict the noise present in the image, along with a fixed variance schedule simplifying the training process. A normal distribution requires the mean and the variance, but the authors decided to fix the variance. This paper employed a linear schedule, wherein the amount of noise in the image increased linearly through the iterations.

For the architecture, the authors used a modified version of the UNet Architecture [14] by Ronneberger, Fischer, and Brox which was originally introduced in 2015 for biomedical image segmentation but, in diffusion models, is used for noise prediction. Broadly, it includes two stages, namely, the encoder and the decoder, wherein it takes in an image as an input and, using downsampling and residual blocks [15], projects the image to a smaller resolution. After reaching the bottleneck, it iteratively projects the image back to its original size, this time using upsampling blocks instead. At certain resolutions, the authors used attention blocks introduced in [16] and employed skip connections between layers of the same spatial resolutions.

The UNet model remains the same for each timestep and iteration, and a sinusoidal embedding is used which decodes the current timestep information to pass to the model. This embedding is projected into each residual block. This is important because the forward diffusion process is performed using a schedule, which scales the mean and the variance. As a result, different amounts of noise is applied at different timesteps, and using this information, the model learns to remove different amounts of noise at different timesteps, which greatly benefits the outcome.

The choice of fixing the variance was rethought by OpenAI researchers Nichol and Dhariwal in [17] in 2021, and they eventually decided to learn the variance too, because it led to improvements in the log likelihood. The authors also found the linear schedule to be suboptimal, as the information gets destroyed too fast, and that the images at the last couple of timesteps already seem to be complete noise, which makes it redundant. Therefore, they introduced the cosine schedule, which was designed to be linear in the middle region and to have little change at the extreme points at the beginning and at the end of the timesteps. This schedule behaves better and takes more time to destroy the information in the image. They also used the denoising diffusion implicit model (DDIM) [18] approach during the inference procedure and found that it produces faster samples with fewer than 50 sampling steps, although the image quality seemed to take a hit in certain cases.

Later in the same year, the same authors Nichol and Dhariwal in [6] claimed to have achieved better image quality using diffusion models as compared to GANs, which had been the most popular generative model being used up to that point. It improved sample quality by using classifier guidance [19], a simple method for trading off diversity for fidelity using gradients from a classifier. It achieved better scores in terms of Fréchet inception distance (FID) when compared to GANs on ImageNet [20]. They heavily improved the overall outcome and the quality of images generated by improving the UNet architecture. Some of the changes they introduced are as follows:

1. Increased the depth of the network and decreased the width.
2. Included more attention blocks and increased the number of attention heads.
3. Used attention at 32 × 32, 16 × 16, and 8 × 8 resolutions, rather than only at 16 × 16.
4. Used the residual block from BigGAN [21] for upsampling and downsampling blocks.
5. Used an adaptive group normalization (AdaGN), where timesteps were incorporated slightly differently.
6. Used classifier guidance.

The efficacy of diffusion models lies in its unique approach to generating images by modeling the iterative application of noise to an initial image, gradually transforming it into the desired output. They were found to demonstrate superior performance to GANs by leveraging this sequential process, enabling them to capture complex data distributions effectively. They have the capacity to handle diverse data types and produce high-fidelity images, while mitigating common issues such as mode collapse and training instability, which showcased their efficiency and potential for advancing image synthesis methodologies.

The sampling speed of these diffusion models was extremely slow, which immensely limited them from being used in any capacity in the real world. There has been quite a lot of research conducted, to try and decrease this sampling speed, by using various methods. For example, Rombach et al. in [1] used a technique where the image formation process is decomposed into a sequential application of denoising autoencoders. Additionally, they introduced crossattention layers into the model architecture which further enhanced the sampling process. Salimans and Ho [22] also attempted to make the sampling speed more efficient using a technique they termed as progressive distillation. In this, a trained deterministic diffusion sampler is distilled, using multiple steps, into a new diffusion model. This distillation process is progressively applied to the model, halving the required sampling steps each time it is applied. Consistency model is a new family of models introduced by Song et al. in [23], which works by directly mapping noise to data. It uses a probability flow (PF) ordinary differential equation (ODE) which smoothly converts data to noise, using which it learns to map any point on the ODE trajectory to its origin for generative modeling. This method outperformed existing distillation techniques for one-step and few-step sampling and set a new benchmark in terms of speed.

Diffusion models were later found to be quite useful in anomaly detection techniques, primarily in the medical domain, and there have been various techniques that have been introduced over the years. GAN-based methods, such as AnoGAN [12], use a scoring mechanism to create an iteratively refined generator approximation; however, this process involves numerous backpropagation iterations, leading to slow inference times. Wolleb et al. in [13] used a novel weakly supervised anomaly detection method based on DDIMs to detect brain tumors. They combined the iterative noising and denoising techniques with classifier guidance [19], in order to be able to differentiate between healthy and nonhealthy subjects and generate anomaly maps. This resulted in a detail-consistent image-to-image translation without the need to change the architecture or training procedure. Pinaya et al. in [24] also used diffusion models in anomaly detection, specifically in brain anomaly detection and segmentation. They propose a method which trains the models on healthy data and then explores its diffusion and reverse steps across its Markov chain to identify anomalous areas. However, inference speed was still a challenge that had not yet been resolved.

All methods that previously used diffusion models for anomaly detection used the default Gaussian noise as the noise function. The idea of using a simplex noising procedure over Gaussian noise in the diffusion process was originally introduced in 2022 by Wyatt et al. in AnoDDPM [25], where the authors found it to perform better at reconstructing healthy images. This approach worked particularly well in reconstructing brain MRIs using simplex noise, over Gaussian noise. In the case of anomaly detection where the goal is to get rid of the anomalous regions in the reconstructed image, Gaussian diffusion failed to capture larger anomalies, due to which AnoDDPM used a multiscale simplex noise diffusion process that gives control over the target anomaly size. It allowed the authors to precisely control the distribution of frequencies present in the image. When simplex noise is used, the corruption is more structured and the denoising process is able to repair those structured anomalies.

Simplex noise was originally derived from Perlin noise [26] but is faster to generate and requires lower computational complexity, though they both visually look the same and include the same parameters. Instead of using the default simplex noise function, Wyatt et al. applied several octaves of noise. This involves combining *N* frequencies of noise together, where the next frequency amplitude reduces by some decay rate *γ*. They found that low-frequency noise cannot be well approximated with a Gaussian distribution; however, by applying an increasing number of octaves of noise, distribution becomes closer to a Gaussian distribution, which is necessary for DDPM as we assume that our noising function is sampled from a Gaussian distribution. The parameters used by the authors when generating simplex noise are as follows:

1. Frequency (*υ*) = 2^−6^
2. Octave (*N*) = 6
3. Decay rate (*γ*) = 0.8

AnoDDPM uses a partial diffusion strategy which uses a shorter Markov chain as compared to a full diffusion process, which results in faster inference and training. However, the speed of inference is still a major limitation for its use in real-world scenarios. We build on top of their work to introduce a faster sampling procedure where we denoise the entire image using a single step, wherein the speed of inference always remains constant since there is no Markov chain present.

## 3. Materials and Methods

A denoising diffusion model consists of two processes, both of which are performed iteratively.

1. Forward diffusion process is used to go from a normal image to complete noise which follows a normal distribution. This is a fixed process and is denoted by *q*.
2. Reverse diffusion process is used to go from complete noise back to an original image. This is a learned process, denoted by *p*_*θ*_, and the model follows a UNet architecture.

The forward diffusion process in DDPM is denoted by *q*(*x*_*t*_|*x*_*t*−1_); it gradually corrupts data from some target distribution *q*(*x*_0_) into a normal distribution. A learned reverse process denoted by *p*_*θ*_(*x*_*t*−1_|*x*_*t*_) generates samples by turning noise into samples from *q*(*x*_0_).

An image is denoted by *x*, with a subscript determining its current timestep, denoted by *t*. For example, an image after adding 250 iterations of noise will be denoted by *x*_250_, and the final image, which will be an isotropic Gaussian, will be denoted as *x*_*T*_. A *T* value of 1000 was empirically found to be the most effective value according to most papers on diffusion.

The iterative forward diffusion process is a fixed process and is denoted by *q*. This process simply takes in an image and returns the image with a little more noise added to it. The amount of noise added at each timestep is defined by a variance schedule *β*_*t*_ *ϵ* (0, 1), *t* = 1, ⋯, *T*, which could be linear, quadratic, cosine, etc., and its goal is to ensure that the data is being scaled in such a way that the variance does not explode. We define *β*_start_ = 0.0001 and *β*_end_ = 0.02 as given in [5] by Ho, Jail, and Abbeel. During forward diffusion, we simply scale down the image iteratively, which acts as a counterpart for increasing the variance over time and keeping the variance in bound. It is a nonhomogeneous Markov chain, meaning that the dynamics of the process can be described by the one-step transition density:

(1) $$\begin{array}{c} q\left(x_{t}\left|x_{t-1}\right.\right)=N\left(x_{t};\sqrt{1-\beta_{t}}x_{t-1},\beta_{t}Ι\right) \end{array}$$

This is the procedure to apply a single forward step to go from *x*_*t*−1_ to *x*_*t*_. To apply multiple forward steps, we could simply repeat Equation (1) multiple times, but this is time-consuming and takes a lot of compute resources due to which we use a faster and more efficient way to perform the forward operation in a closed form to go from *x*_0_ to any arbitrary timestep in a single step. Corresponding to this, we introduce some more notations for *α*_*t*_ and its cumulative product $\bar{\alpha_{t}}$ in Equations (2) and (3), respectively:

(2) $$\begin{array}{c} \alpha_{t}=1-\beta_{t} \end{array}$$

(3) $$\begin{array}{c} \bar{\alpha_{t}}=\Pi_{s=1}^{t}\alpha_{s} \end{array}$$

Using the reparameterization trick, the forward diffusion equation can be rewritten to perform the noising process, where we go from *x*_0_ to *x*_*t*_ using a single step. *ϵ* is the noise sample and is taken from a normal distribution:

(4) $$\begin{array}{c} q\left(x_{t}\left|x_{0}\right.\right)=N\left(x_{t};\sqrt{\bar{\alpha_{t}}x_{0}},\left(1-\bar{\alpha_{t}}\right)Ι\right) \end{array}$$

(5) $$\begin{array}{c} q\left(x_{t}\left|x_{0}\right.\right)=\sqrt{\bar{\alpha_{t}}}x_{0}+\sqrt{1-\bar{\alpha_{t}}}\epsilon \end{array}$$

The reverse diffusion process is denoted by *p*_*θ*_. This is a learned process that is parameterized by *θ*. It takes in an image *x*_*t*_ as input and produces a sample *x*_*t*−1_ using the trained neural network. It simply decreases the total amount of noise present in the input image:

(6) $$\begin{array}{c} p_{\theta }\left(x_{t-1}\left|x_{t}\right.\right)=N\left(x_{t-1};\mu_{\theta }\left(x_{t},t\right),\underset{\theta }{\sum }\left(x_{t},t\right)\right)\; \end{array}$$

In Equation (6), we have two neural networks, the mean (*μ*_*θ*_) and the variance (∑_*θ*_) which parameterize the normal distribution, which we can sample from to get *x*_*t*−1_. We choose to fix the variance to a certain schedule and therefore do not need a neural network for this, and it can simply be replaced by *β*_*t*_. We learn a neural network to predict the noise present in the image (*ϵ*_*θ*_); we simply need to predict the mean (*μ*_*θ*_); this is demonstrated in Equation (7):

(7) $$\begin{array}{c} \mu_{\theta }\left(x_{t},t\right)=\frac{1}{\sqrt{\alpha_{t}}}\;\left(x_{t}-\frac{\beta_{t}}{\sqrt{1-\bar{\alpha_{t}}}}\;\epsilon_{\theta }\left(x_{t},t\right)\right) \end{array}$$

To sample the previous image in the chain *x*_*t*−1_, we apply the reparameterization trick to obtain Equation (8), where *σ*_*t*_*z* is the additional noise function being added to the denoised image, and *z* ~ *𝒩*(0, *I*):

(8) $$\begin{array}{c} x_{t-1}=\frac{1}{\sqrt{\alpha_{t}}}\;\left(x_{t}-\frac{\beta_{t}}{\sqrt{1-\bar{\alpha_{t}}}}\;\epsilon_{\theta }\left(x_{t},t\right)\right)+\sigma_{t}z \end{array}$$

The training objective of the DDPM is to predict the noise present in an image between two subsequent timesteps using a neural network. The loss function used is the mean squared error (MSE) between the actual *μ* and the predicted *μ* and is given in Equation (9). This is simply the difference in pixel values between the actual noise (*ϵ*) and the predicted noise (*ϵ*_*θ*_) by te neural network, given some arbitrary image at timestep *t*. Value of *t* is sampled from a uniform distribution between 1 and *t*:

(9) $$\begin{array}{c} L_{\text{MSE}}={\left\|\epsilon -\epsilon_{\theta }\left(x_{t},t\right)\right\|}^{2} \end{array}$$

### 3.1. Datasets

The nonanomalous (healthy) dataset used is the neurofeedback skull-stripped (NFBS) repository [27]. It consists of manually corrected brain masks for 125 T1-weighted anatomical scans from the Nathan Kline Institute Enhanced Rockland Sample Neurofeedback Study. It is composed of images from individuals with ages ranging from 21 to 45 and represents individuals diagnosed with a wide range of psychiatric disorders. Image resolution is 256 × 192 and contains a total of 256 slices of the brain from the top-down view. For each volume, we have about 60 trainable slices after dismissing the top and bottom few slices of the volume, as these generally do not have the brain structure that we want to train the model on. This puts us at a total of about 7500 brain slices that we use to train the model.

The anomalous (unhealthy) dataset used is provided by Edinburgh University and is titled “A neuroimaging dataset of brain tumour patients” [28], which is a public dataset containing T1 and T2 imaging data from 22 patients. These 3D MR volumes are used during the sampling process to test the model and its capability at detecting anomalies. The image resolution is 256 × 156 and contains a total of 256 slices of the brain from the top-down view, out of which around 30–60 slices are anomalous, giving us a total of a little over 900 slices to test the working of the model on. In the case of anomalous dataset, along with the 3D Brain MR volume, we also have the actual mask (ground truth), which communicates the region where the anomaly is present in the 3D image. This is useful during sampling to evaluate the model to test how accurate it is at detecting large anomalous regions.

In the medical imaging field, data samples are usually in the Neuroimaging Informatics Technology Initiative (NIfTI) file format [29] and is an open file format commonly used to store brain imaging data obtained using MRI methods. We use datasets consisting of 3D MRI volumes for both training and inference. We enhance the contrast of the image by stretching the pixel intensity values so that the darker and lighter parts of the image are spread across a wider range of intensities, making the image easier to analyse. Respective transformation operations are added to the 3D MR volumes and are saved as a NumPy file, so that it can later be accessed without requiring us to repeat any of the transformations. This also makes performing NumPy operations on the images much faster and makes subsequent accesses to the dataset quicker. All images are resized to 256 × 256 before being input to the neural network.

We use axial slices of the brain since anomalies are generally easier to spot from this view. When an image is sampled from the dataset, it selects a random slice from the appropriate range and passes it on to the model. It converts the 3D NumPy array to a 2D array of a particular slice, which in our case is a single channel (black and white) 2D image.

### 3.2. Model Architecture

**Figure 1:**
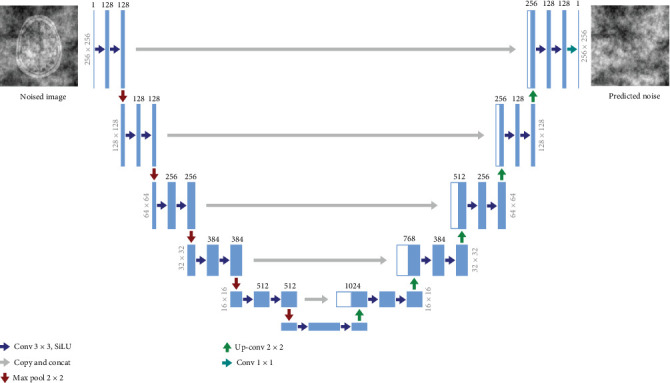
UNet model architecture. Images are 256 × 256 in the highest resolution and 16 × 16 in the lowest resolution. Blue boxes correspond to a multichannel feature map, with the number at the top of the box depicting the number of channels and the number on its left depicting the image resolution at that level. White boxes represent feature maps, obtained through skip connections, which are concatenated in the upsampling phase. The input to the neural network is a noised brain image, and the output is the predicted noise in the image.

The UNet model is used to learn the structure and features of the images in the dataset at a deeper level, while learning to segment and estimate the noise. This is where the learning process happens, and the exact architecture used in our experiments is demonstrated in Figure 1. Our model architecture follows the backbone of PixelCNN++ [30], which is a UNet based on a Wide ResNet [31], with transformer sinusoidal positional embedding to encode the timestep. The image goes through a series of downsampling operations along with residual and attention blocks where the image resolution decreases, while the number of channels increases. It then reaches a bottleneck, after which it moves on to the decoding phase, which is a series of upsampling operations, where the image resolution is gradually increased, while the number of image channels decreases back to its original format. Essentially, it processes a given image by progressively halving the feature map resolution in the first half and then progressively increasing the resolution in the second half of the process, helping it to learn more features in the process. There are pass-through skip connections at each resolution, coming in from the encoding phase into the decoding phase.

The input to the neural network is a noised brain image, and the objective of the UNet is to separate the noise from the actual brain image. The output image of the model will have the same dimensions as the input image but will simply be an image consisting of the noise estimate, completely getting rid of the original image signal.

### 3.3. Training

An image sampled from the healthy dataset for training simply receives a random 2D image slice from the 3D MR volume, after which a random timestep value is picked which may be anywhere between 0 and 1000. The objective of the model is to estimate the noise at any timestep in this region. Then, a simplex noise sample is obtained. We now have three separate elements, the original brain image, a noise sample, and a timestep value. We then apply the forward diffusion process from Equation (5) to add this noise to the image using a single step, which consequently gives us a noised version of the original brain image from the dataset.

The training objective is to estimate the noise present in the given image. As demonstrated in Equation (9), the loss function will be a simple MSE between the actual noise sample added to the image and the noise estimated by the model. The simplex noise function we use always creates a random noise image with random artifacts, and the timestep value is also random, due to which the model can efficiently learn what constitutes as the noise in an image at various different noise levels.

**Figure 2:**
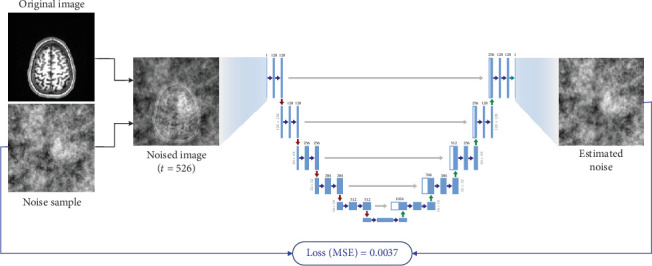
Single training iteration. Images are noised up to a random timestep value (*t*) and passed as input to the neural network, which then learns to estimate the noise present in the image. Loss is calculated by finding the MSE between the actual noise (*ϵ*) and predicted noise (*ϵ*_*θ*_).

Figure 2 showcases a single iteration of the training procedure. A single iteration involves sampling an image from the dataset, adding noise to it, and passing it through the neural network architecture, after which the estimated noise output is compared with the original noise sample created using MSE. This loss value is then backpropagated to the nodes and weights are modified.

By around 100,000 iterations of the healthy dataset on the model, it efficiently learns to estimate the noise and also indirectly learns the underlying structure of the original image as a secondary objective. This now allows the model to create completely original brain images of its own, by just attempting to remove the noise present in a noised image, since the UNet model has now learnt the latent representation of the brain images present in the dataset.

### 3.4. Sampling

The main goal of sampling is to reconstruct a healthy brain image from an unhealthy brain image, so that we can then look for pixel-level differences between the images and pinpoint the anomaly. When an image is sampled from the anomalous dataset, we receive a 2D image of a particular slice from the 3D volume and additionally also receive the ground-truth anomaly mask of this slice, which is required for evaluation of model accuracy during testing. Like in the training process, a simplex noise sample is created, and a random timestep value is picked.

**Figure 3:**
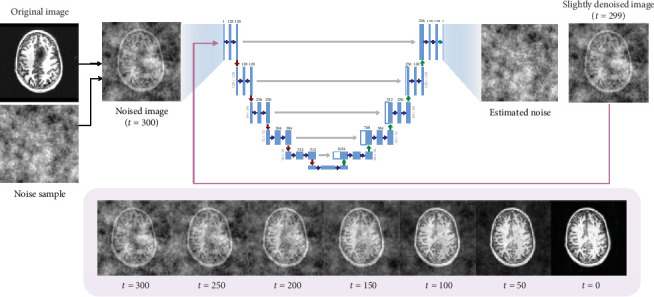
Iterative sampling procedure used in DDPM [17]. The reverse diffusion process takes place iteratively, where in each iteration of the neural network, we only remove a small amount of the predicted noise from the input image and subsequently send the image back to the neural network as input. This operation is performed repeatedly until *t* = 0, at which point we obtain the newly constructed image.

While sampling, the range of acceptable timestep values is significantly smaller. The usage of a partial diffusion process wherein the image is only slightly noised rather than fully noised is an extremely essential step in anomaly detection using diffusion models, as we do not want to fully get rid of the original image signal. This is so that a general structure of the image is still visible, and the model has some idea as to what kind of brain image it must create. The timestep value we found to work best for our use case is between 250 and 400, depending on the size of the anomalous region. The noise sample is then added to the original image at a particular timestep using the same forward diffusion process used in training, after which it is passed as input to the UNet model. The trained neural network then estimates the noise present in the image, using the data samples it learned during training.  Figure 3 demonstrates the iterative sampling procedure introduced in DDPM, where denoising takes place in an iterative fashion where we remove a small amount of noise from the image in a guided manner, in order to obtain a high-quality image by the end.

Once we have obtained the reconstructed brain image, denoted by ${\hat{x}}_{0}$, we simply perform a pixel-wise comparison of the two images, the original and the reconstructed as given in Equation (10). *E*_*sq*_ denotes the MSE between the images:

(10) $$\begin{array}{c} E_{sq}={\left(x_{0}-{\overset{\wedge }{x}}_{0}\right)}^{2} \end{array}$$

This operation generates a heat map, demonstrating which areas in the reconstructed image are most different from the original image. The areas with the highest intensity differences are then considered the segmented anomaly. We set a naïve threshold value of 0.3 and convert it into a binary image, which will be the resulting anomaly mask. The resulting mask is then compared to the ground truth, and its performance is evaluated using segmentation evaluation metrics such as intersection over union (IoU) and the Sørensen–Dice coefficient.

The issue with this reverse diffusion approach is that it is a very time-consuming and compute-intensive process, even on the highest-end GPUs available. For example, if an image was noised up to 400 timesteps, then it must go through that many iterations of the reverse diffusion process, wherein the noised image is passed through the neural network 400 times, as it slowly and iteratively removes a small amount of noise in each timestep, until it reaches *t* = 0, where we obtain a fully denoised image. Thus, we come to our improved sampling approach, which is aimed at increasing the speed while still obtaining comparable reconstructions of the brain image.

We observe that during the iterative reverse diffusion process of the DDPM sampling approach used in [25], since the model has only been trained to estimate the noise present in an image at once, when it attempts to remove a small amount of noise for a particular timestep, say *t*, what actually happens is that it first removes all the noise present at that particular timestep, obtaining an intermediate image that the model is slowly inching towards, and then adds most of the noise back to it, getting us the image at the previous timestep  *x*_*t*−1_. This operation takes place *t* times, until it reaches *t* = 0 which will be our newly reconstructed image.

**Figure 4:**
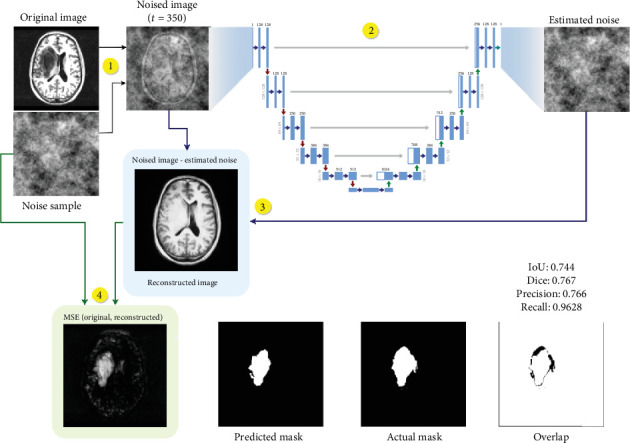
Single-step sampling procedure.

Through our experiments, we found that while sampling from an anomalous image during the reverse diffusion process, rather than removing noise from the noised image iteratively until *t* = 0, we can simply remove all the noise that is present in the original noised image within a single step and not add any of the noise back, bringing us from *x*_*t*_ to *x*_0_ within a single iteration of the neural network. This is demonstrated in Algorithm 1, and it works well for our use case of anomaly detection since we simply want the anomalous regions to be repaired while the rest of the image retains its structure, without giving much importance to image quality.  Figure 4 describes the SSS procedure using the following four steps:

1. An image is sampled from the dataset, and a simplex noise sample is created. The noise is added to the image at any random timestep between 250 and 400.
2. Noised image is passed through the UNet model which goes through a series of downsampling and upsampling operations and outputs an estimated noise image.
3. Remove this estimated noise from the original image which was input to the model, in order to get the reconstructed brain image, which ideally gets rid of the anomaly.
4. Find the MSE between the original and the reconstructed images, giving us the pixel-level difference between images. This leads us to our final predicted mask, which can then be compared with the ground truth in order to determine the accuracy of our prediction.

There are a few reasons why this method of sampling works specifically in case of anomaly detection:

1. Since we only apply a partial diffusion process, a general structure of the original image signal is still visible, due to which the trained model can remove all the noise present in the noised image in a single step while still maintaining comparable image quality.
2. The dataset consists of a single class of images where the structure of brain images remains mostly the same, so the model can confidently guess what the brain region behind the noised patches might look like, getting rid of the anomaly in the process. Therefore, this sampling technique is particularly useful in case of one-class anomaly detection.
3. Since the images are in greyscale, and so is the simplex noise function, the model mistakes the noise regions to be a part of the anomaly and gets rid of these areas while attempting to reconstruct the brain image, replacing it with healthy brain tissue.

**Figure 5:**
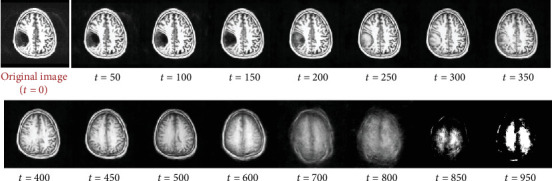
Reconstructed brain images after single-step sampling from various timesteps. Note that at extremely high timestep values, the model has lost most of its features and is nowhere near the image quality that we require; therefore, it is not recommended to exceed the range of ideal timestep values. We also note that reconstructing from anywhere below 200 timesteps does not repair the existing anomaly.

While sampling, we need to make sure that the timesteps are anywhere between 250 and 400. The image quality will be significantly reduced if the sampling timestep is anywhere over this range, thus resulting in inaccurate anomaly masks, as the reconstructed image obtained will barely resemble the original image owing to its high timestep value which destroys the original image signal. We also need to make sure that the timestep value is not too low, as this will not be enough to get rid of the anomalous region in the reconstructed image.  Figure 5 demonstrates the reconstructed images after their single-step reconstruction after they are noised at different timestep values.

In terms of anomaly size, we generally notice that higher timesteps are used to get rid of larger anomalies, while smaller timesteps are enough to get rid of anomalies of smaller size, though it is still important for the timesteps to be higher than 200.

**Figure 6:**
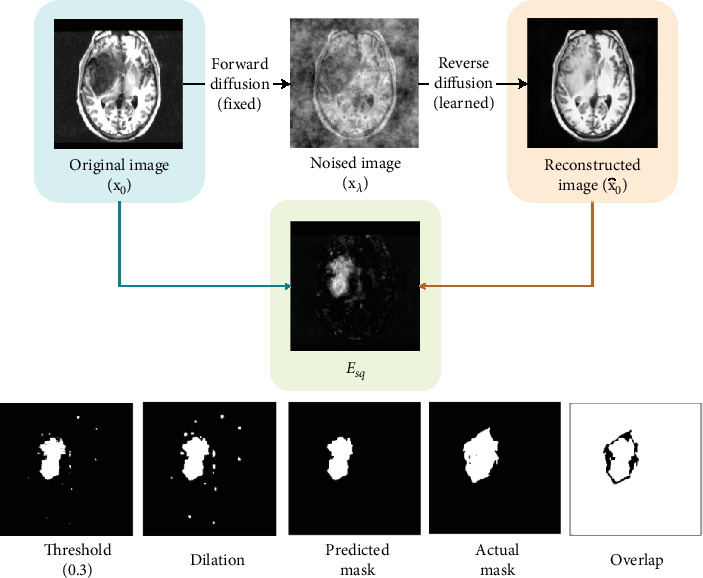
Postprocessing operations.

Once the MSE between real and reconstructed images is obtained, we may find that there are little, tiny regions that are incorrectly detected as an anomaly, which might be detrimental to the final evaluation metrics that we use to compare to the ground truth. Therefore, we attempt to remove any inaccurate anomalous regions that may have been created using sufficient postprocessing operations. We perform a dilation operation with a kernel size of 3 × 3, which expands the area of the detected anomaly. We also get rid of all the smaller regions and only keep the largest anomalous regions present in the image in terms of area. This way, we only keep the most significant anomaly masks that are obtained from the model. This has a minimal but nonnegligible impact on the final IoU (intersection over union) and Dice values. Postprocessing operations applied are demonstrated in Figure 6.

### 3.5. Experimental Setup

We employ various techniques to enhance the training process of our deep learning model. An exponential moving average (EMA) is incorporated to stabilize the model's convergence and alleviate the effects of noise. Specifically, we set the beta decay rate of the EMA to 0.9999, which allowed the model to retain information from a long history of gradients. This approach facilitated a more robust and consistent learning process. Additionally, we utilized the AdamW optimizer [32], a variant of the Adam optimizer, which includes weight decay regularization. The AdamW optimizer has demonstrated improved performance in various deep learning tasks. We set the learning rate to 0.0001, enabling fine-grained adjustments during training, and the beta parameters were configured to 0.9 and 0.999, respectively, to control the exponential decay rates of the first and second moment estimates.

These choices were made based on prior research and empirical evidence suggesting their effectiveness in optimizing deep learning models. By employing the EMA with a beta decay rate of 0.9999 and the AdamW optimizer with a learning rate of 0.0001 and betas of 0.9 and 0.999, we aim to enhance the model's training stability and convergence speed.

After appropriate hyperparameter tuning, the values we found to perform best on the brain MRI dataset are given in Table 1.

**Figure 7:**
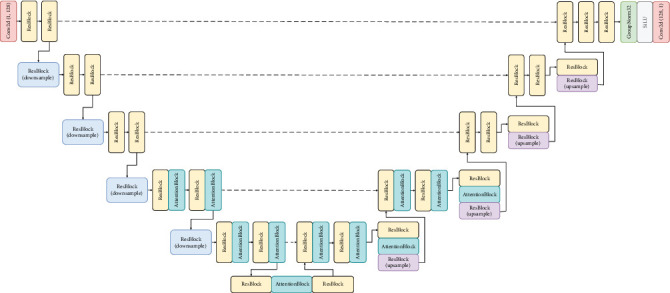
Detailed UNet [14] architecture.

Figure 7 demonstrates the UNet architecture in detail, including the residual and attention blocks. We can see that attention blocks have been used in the last two levels of UNet where image resolutions are 32 × 32 and 16 × 16.

The code for this paper was implemented in Python using PyTorch. We used open-source implementations from various GitHub repositories, including the guided diffusion implementation by OpenAI, UNet architecture used in DDPM, and simplex noise by OpenSimplex. Training for the diffusion model took around 17 h and was performed on a single NVIDIA A100 GPU with 40 GB RAM.

## 4. Results and Discussion

The accuracy of the predicted mask is evaluated by comparing it with the actual mask using segmentation metrics such as IoU and Dice. These are the most useful indicators for checking how accurate a predicted mask is compared to the actual mask. An IoU value of 1 is said to have a perfect match, whereas a value of 0 would mean that it did not predict any of the mask areas correctly. Furthermore, metrics such as precision and recall also play a role in determining the accuracy of the predicted mask.

**Figure 8:**
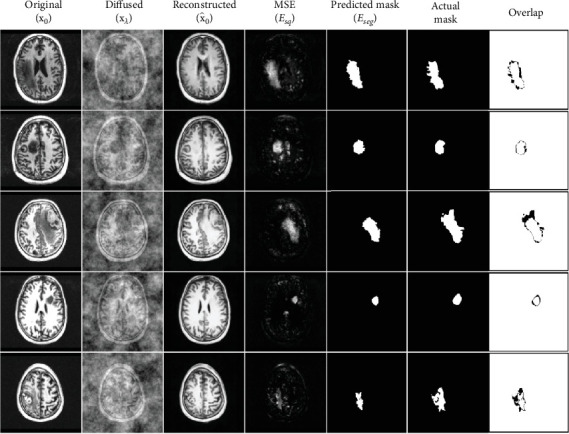
End-to-end sampling anomaly detection and segmentation procedure. Diffused images have been noised up to 300 timesteps, wherein *λ* = 300. *E*_*sq*_ is the mean squared error between the original and reconstructed images, calculated using $E_{sq}={\left(x_{0}-{\overset{\wedge }{x}}_{0}\right)}^{2}$. *E*_seg_ is the final predicted anomaly mask. The final column is simply the actual and predicted masks overlapped on top of each other for visualization purposes.

For our sampling procedure, we pick four equidistant slices that are within the range of anomalous regions in the 3D volume. The anomalous slice is noised and passed to the trained model to reconstruct it into a healthy brain image. The reverse diffusion process successfully denoises this image, creating a healthy image in the process. Since the model was only trained on healthy images, the reconstructed sample will have repaired the anomalous region. The output of the model will simply be a reconstruction of the original brain image.  Figure 8 demonstrates a few examples of image reconstructions and their respective segmentation masks as compared to the ground truth.

To obtain the segmentation mask, we find pixel-level differences between the original and reconstructed images and convert the subsequent image into a binary mask given a threshold value. This gives us our resulting anomaly mask which can then be compared with the ground truth to evaluate its accuracy.

**Figure 9:**
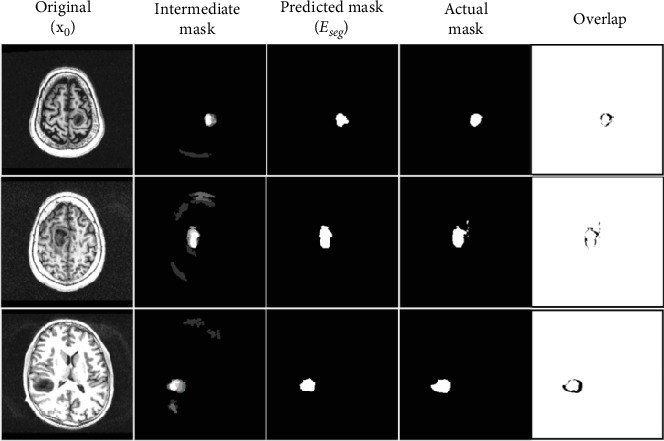
Alternative inference technique. Original image is reconstructed after being noised up to four different timesteps (250, 300, 350, and 400), resulting in multiple brain reconstructions. Predicted anomaly masks are obtained and then overlapped on top of each other to locate pixels that are most commonly detected as an anomaly among multiple reconstructions. After this, a naïve threshold value is picked to finalize the anomalous region.

In [25], the authors suggested that since the algorithm behaves stochastically, future work could explore sampling an image more than once at multiple different timestep values and averaging the corresponding reconstructions based on which pixels are most frequently detected as an anomaly. Since the sampling speed is now just a fraction of the original sampling speed, we perform this improved inference technique without experiencing much overhead. We run the same sampling process multiple times over different timesteps and take the most frequent pixels of the segmentation mask to be the predicted anomalous regions in the brain image. We observe a slight improvement in IoU and precision metrics when using this alternative inference technique, as demonstrated in Figure 9.

However, it was noted that when this alternative technique is used, even though we are more likely to accurately locate the anomaly, it might slightly modify the shape of the resulting anomalous region, negatively affecting the Dice value, but this does not affect the other metrics.

In terms of the size of the anomalous regions that the model is able to detect, we find that the diffusion model is great at detecting large anomalous regions but usually misses out on smaller regions. This is likely due to the parameters of simplex noise used such as octaves, persistence, and frequency, which incentivizes it to mainly focus on larger anomalous regions. In future work, these values could be experimented with to empirically determine the best simplex noise parameters to accurately detect anomalous regions of all sizes.

Table 2 showcases the metrics we achieve from using the iterative sampling method and our SSS approach.

Our experiments presented us with a slight improvement over the previous anomaly detection using diffusion model methods in terms of Dice value, but it has a significant improvement in the IoU, precision, and recall metrics as compared to previous methods due to the postprocessing operations performed. Overall, we see an improvement of 66.3% in the IoU value, 35.3% in the precision value, 24.2% in the recall value, and 18% in the area under the curve (AUC) value. We infer that our SSS approach was successfully able to detect the presence of an anomaly and its general location 80% of the time.

However, the main improvement made by our findings is by far in the speed of the sampling process, which decreases the speed significantly as compared to AnoDDPM, since sampling is now performed in a single iteration of the neural network architecture rather than traversing through the network hundreds of times to generate a single image. For example, if we want to sample from an image noised up to a particular timestep, say *λ*, where it would previously take *𝒪*(*λ*) time to sample the image, when we use our proposed SSS procedure, we can perform sampling in a constant time of *𝒪*(1) regardless of the *λ* value. In terms of clock time, we see that our method reconstructs images at a speed of over 250 times the original speed.

**Figure 10:**
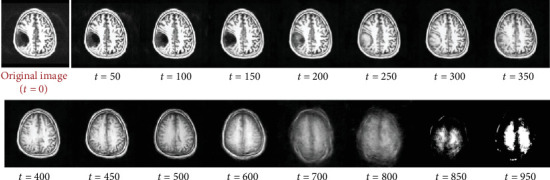
Reconstructed images after single-step sampling from various timesteps (*t*).

Since we use a SSS approach where we expect the trained model to predict the entirety of the brain image within a singular step, in order to be able to get an image where the anomaly has been repaired while still being of considerable quality with no additional artifacts in the reconstructed image, we require the timestep value, or the amount of noise added to the image, to be within a given limit. Generally, we notice that the model performs best at any timestep in the range of 200–350, as denoted in Figure 10. Note that at extremely high timestep values, the model still has a rough idea of the image but has lost most of its identifiable features and is nowhere near the image quality that we expect to be able to compare it with the original image. Therefore, it is not recommended to exceed the range of ideal timestep values.

## 5. Conclusions

In this work, we demonstrated a method in which, given an anomalous brain image, we can reconstruct a healthy version of the image using a single forward and reverse diffusion process, rather than using the usual iterative reverse process demonstrated in DDPM, which takes a lot of clock time and compute resources. This faster sampling approach allows us to perform the reconstruction process on multiple slices of a 3D MR volume at a faster speed, which could eventually be used in clinical scenarios where a radiologist might not have time to go through large quantities of brain image slices. Specifically, this method of reconstruction can be used in one-class anomaly detection where the sole purpose is to reconstruct an image without the anomaly, so that we can find pixel-level differences between the reconstructed and the original image in order to obtain the segmentation mask. For example, our proposed sampling technique could be used in various other domains such as plant disease detection, manufacturing quality control, environmental monitoring, and other medical diagnosis.

The reconstructed image in its current form and architecture may be able to detect and repair large anomalous regions in the brain image but is not always able to detect lesions that are very small, usually leaving these areas intact. Future work could be done in this regard, perhaps by modifying the model architecture or by tweaking the parameters involved with the simplex noise function, which could help train the model to detect even smaller artifacts and learn their structure in the original image.

In this approach, we used a simplex noising procedure rather than the usual Gaussian noise used in diffusion models, which seemed to work better for brain images. Different parameter permutations for octave, persistence, and frequency could be experimented with to find the best fit. Subsequently, more diverse noising functions could be explored such as Poisson noise and speckle noise, which might work better for certain use cases and datasets.

## Figures and Tables

**Algorithm 1:**
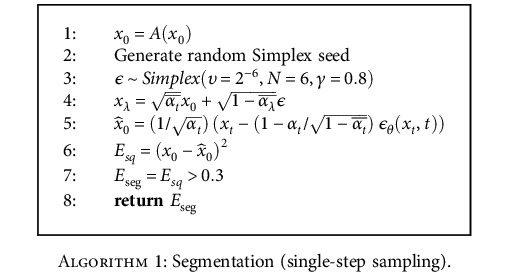
Segmentation (single-step sampling).

**Table 1:** Model hyperparameters.

Epochs	1500
Training slices	7500
Batch size	1
Image size	256 × 256
Beta schedule	Linear
Diffusion timesteps	1000
Loss type	L2
Loss weight	0
Learning rate	0.0001
Weight decay	0.0
Dropout	0
Optimizer	AdamW
*β*_1_, *β*_2_ (AdamW)	0.9, 0.999
EMA rate (beta decay)	0.9999
**Base channels**	**128**
**Channel multiplier**	**1, 1, 2, 3, 4**
**Residual blocks**	**2**
**Attention heads**	**2**
**Attention resolutions**	**32, 16**

*Note:* Rows with bold entries modify the architecture of the UNet model.

**Table 2:** Segmentation performance for a threshold of 0.3 on the anomalous dataset. AnoDDPM uses the iterative sampling approach, whereas SSS uses the proposed single-step sampling approach. Timestep values used for comparison are 250 and 300. Dice, IoU, precision, and recall are used as metrics for segmentation and AUC as classification metric. Time taken for the segmentation procedure for a single image is denoted in seconds. For our experiments, we sample four equidistant slices from each of the brain volumes from the anomalous dataset and average the computed metrics for each slice.

	**Dice**	**IoU**	**Precision**	**Recall**	**AUC**	**Time (s)**
AnoDDPM *t* = 250	0.463 ± 0.209	0.339 ± 0.178	0.446 ± 0.221	0.574 ± 0.269	0.781 ± 0.133	50.494
AnoDDPM *t* = 300	0.411 ± 0.193	0.285 ± 0.162	0.428 ± 0.22	0.455 ± 0.241	0.721 ± 0.119	60.916
AnoDDPM *t* = 250 (with postprocessing)	0.468 ± 0.223	0.454 ± 0.227	0.554 ± 0.25	0.681 ± 0.306	0.836 ± 0.152	88.832
AnoDDPM *t* = 300 (with postprocessing)	0.394 ± 0.199	0.403 ± 0.245	0.504 ± 0.277	0.615 ± 0.34	0.802 ± 0.169	106.472
SSS *t* =250 (ours)	0.441 ± 0.257	0.455 ± 0.226	0.535 ± 0.255	0.713 ± 0.322	0.851 ± 0.16	0.263
SSS *t* = 300 (ours)	0.466 ± 0.216	0.465 ± 0.219	0.559 ± 0.237	0.711 ± 0.296	0.851 ± 0.148	**0.238**
SSS—common regions (ours)	0.367 ± 0.195	0.474 ± 0.244	0.579 ± 0.271	0.665 ± 0.327	0.828 ± 0.163	1.425

*Note:* Boldface data highlights metrics where the proposed single-step sampling approach outperforms the iterative sampling approach used in AnoDDPM.

## Data Availability

The NFBS Skull-Stripped Repository or the nonanomalous dataset is available at http://preprocessed-connectomes-project.org/NFB_skullstripped/, and the anomalous dataset is available through the UK Data Service under the collection name “A neuroimaging dataset of brain tumour patients,” at https://reshare.ukdataservice.ac.uk/851861/.
